# Supplementary figures and images for: Sex-specific plasticity and the nutritional geometry of insulin-signaling gene expression in Drosophila melanogaster
Source: EvoDevo. 2021 May 14;12:6. doi: 10.1186/s13227-021-00175-0 (PMC8120840; doi:10.1186/s13227-021-00175-0)

A

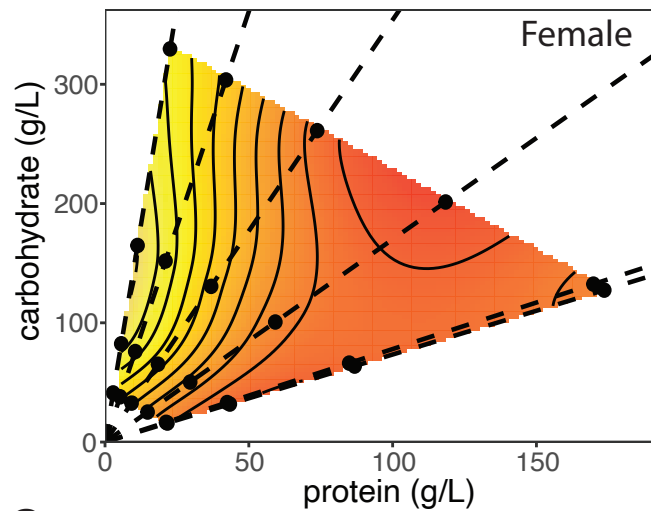

B

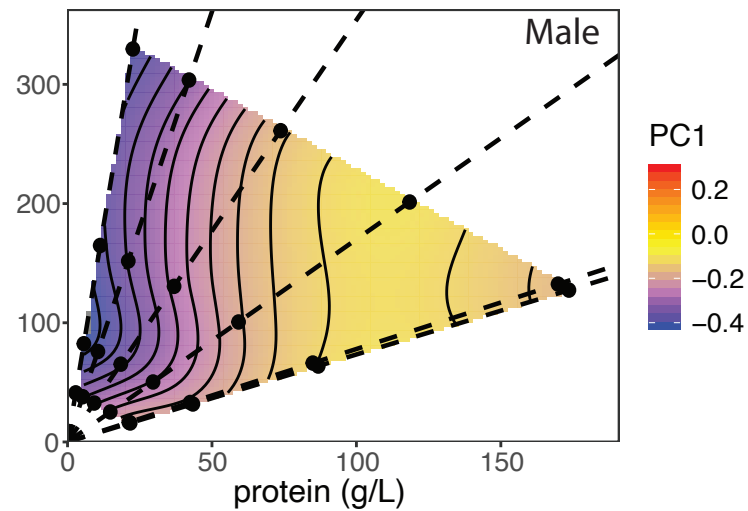

C

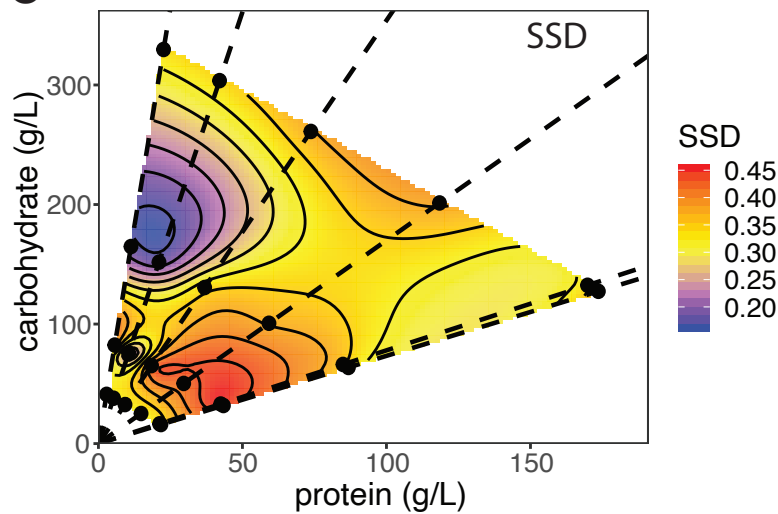

Supplement: Supplementary file 1 — Additional file 1: Fig. S1. Thin plate spline of the effect of protein and carbohydrate concentration on female and male body size and sexual size dimorphism (SSD). (A, B) Surfaces shows the relationship between body size, carbohydrate level and protein level in female and male flies (C) Surface shows a thin plate spline of the difference in female and male body size (SSD) across the same nutritional landscape, using fitted values from A and B. Points indicate diets tested and dotted lines connect diets with equal protein-to-carbohydrate ratios (1:14.6, 1:7.2, 1:3.5, 1:1.7, 1.3:1, 1.4:1). [file 13227_2021_175_MOESM1_ESM.pdf]

♀

A

*lnR*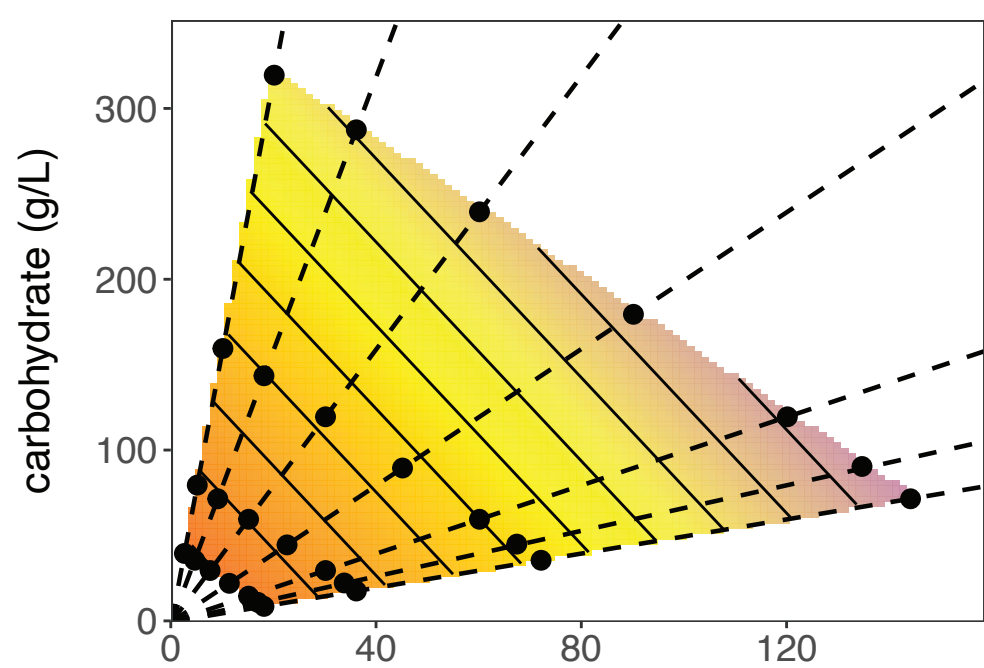

♂

A' *lnR*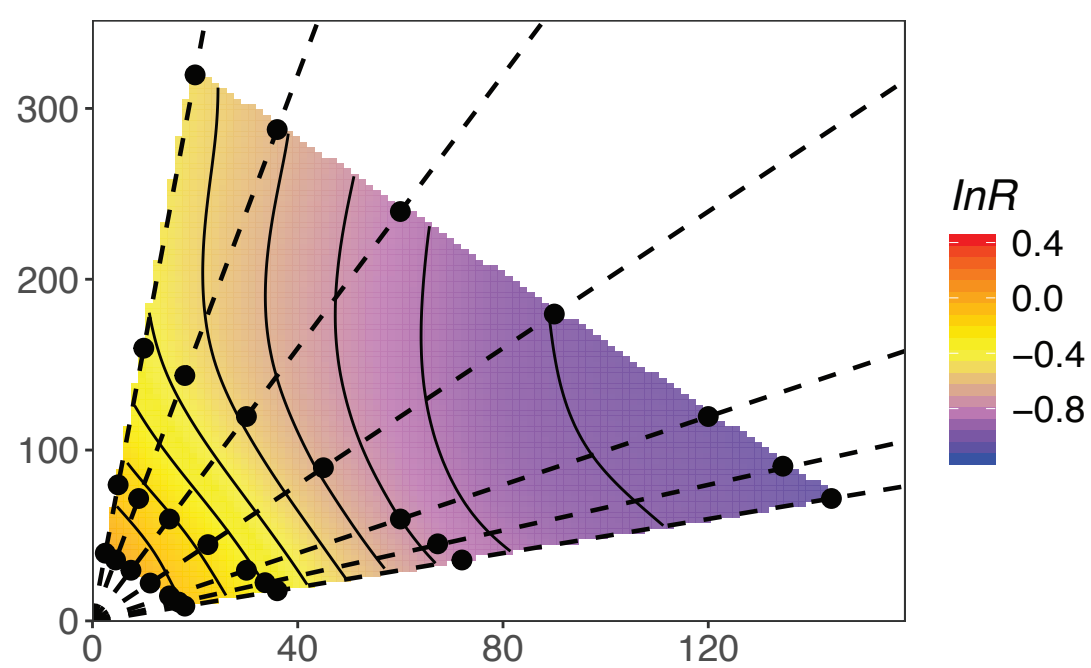

B

*4E-BP*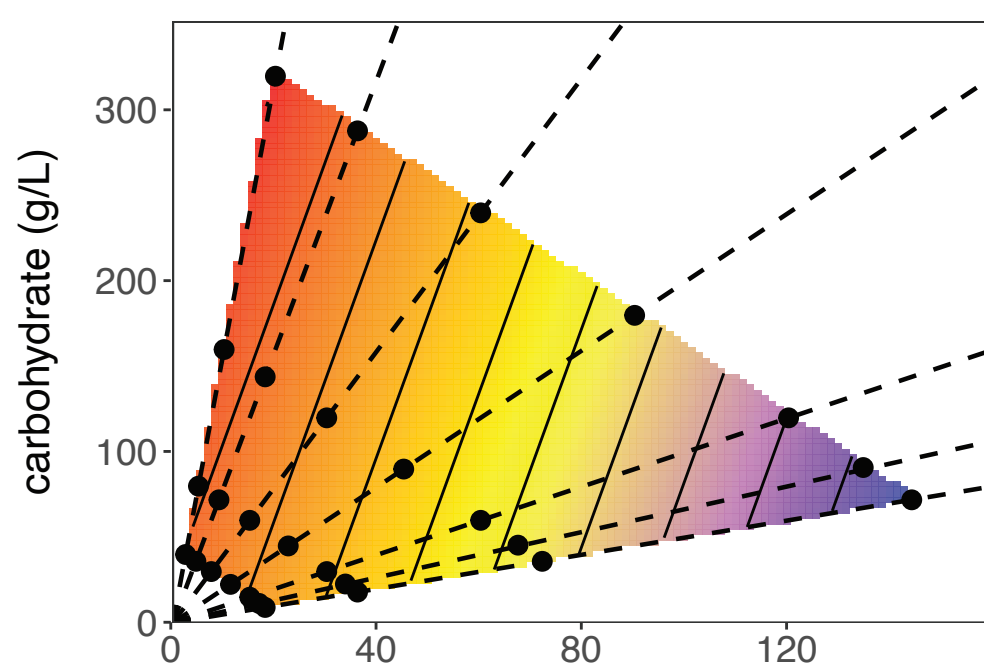B' *4E-BP*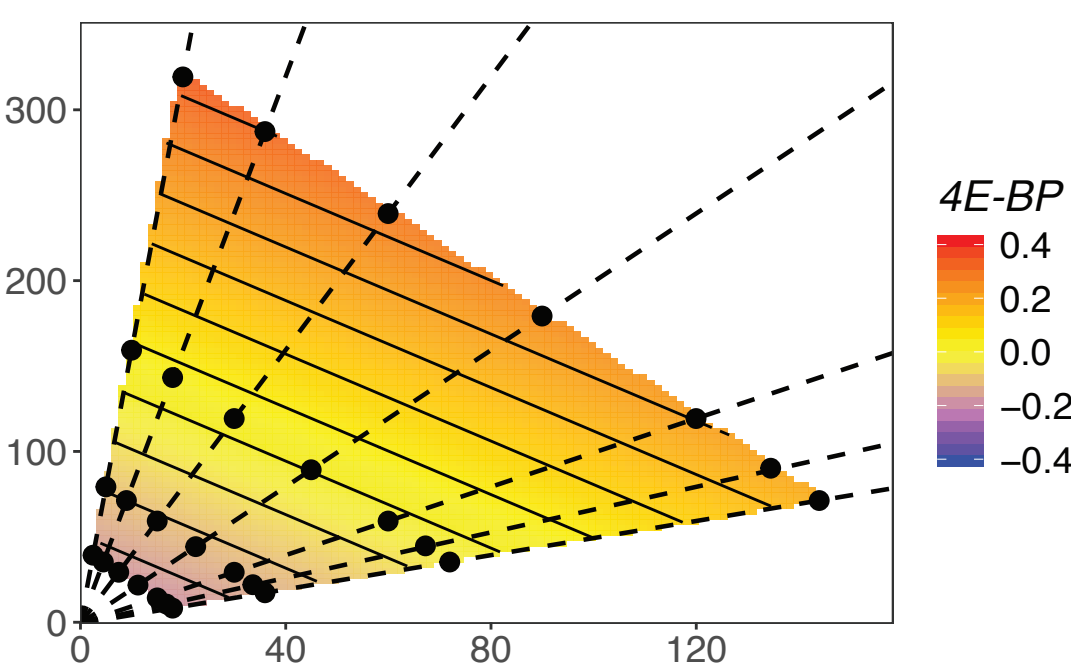

C

*Ash2L*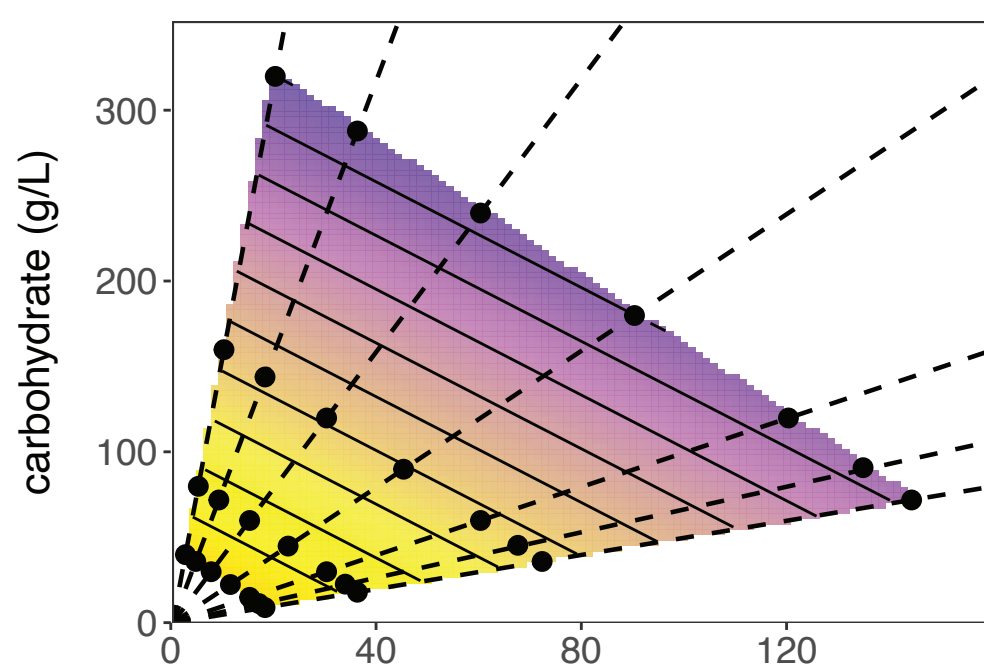C' *Ash2L*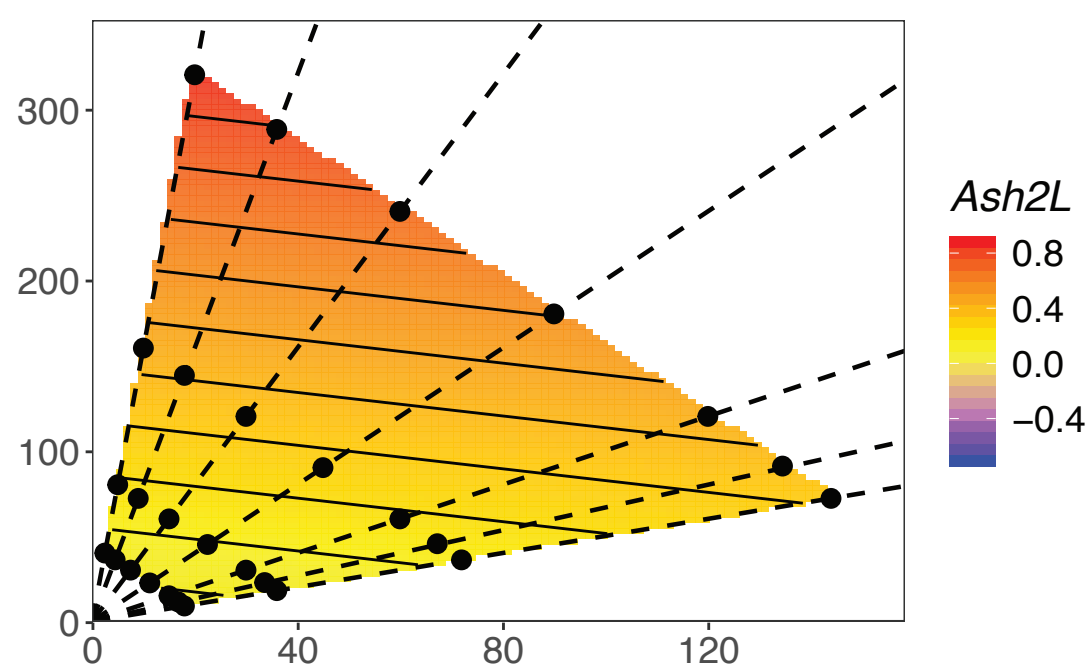

D

*CG3071*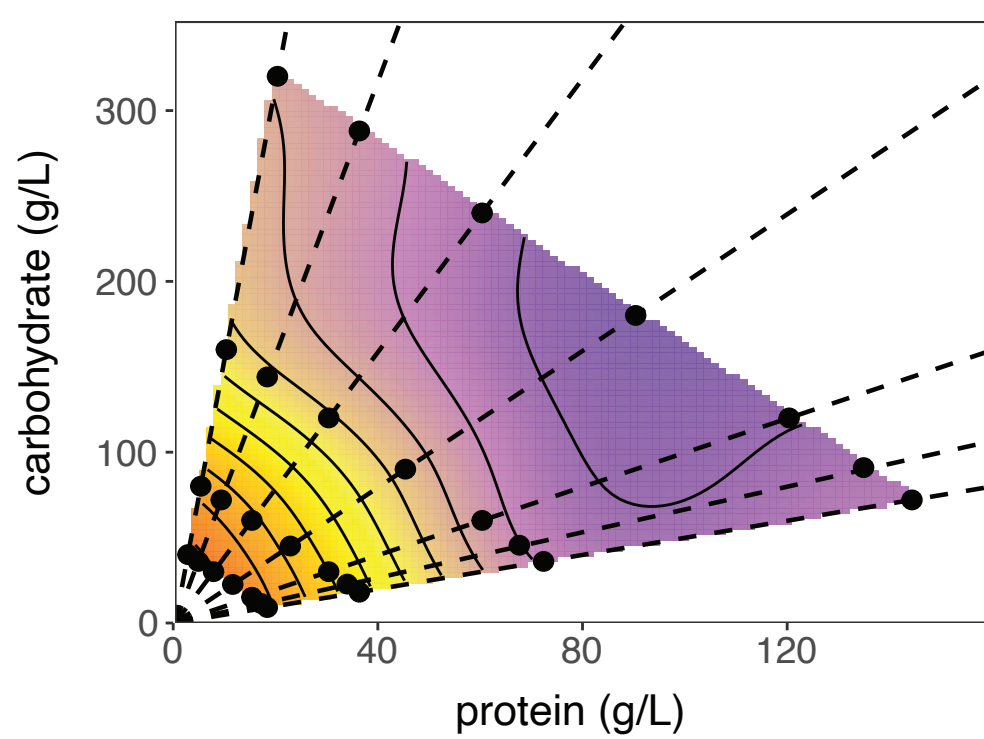D' *CG3071*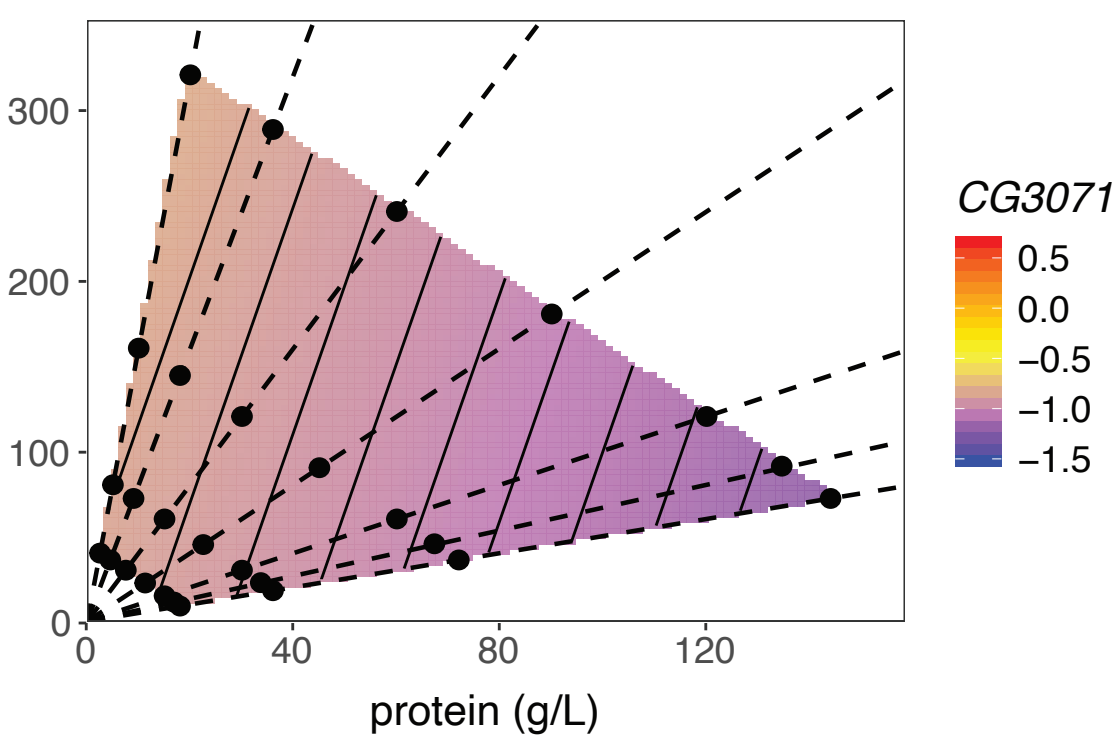

Supplement: Supplementary file 2 — Additional file 2: Fig. S2. Thin plate spline of the effect of protein and carbohydrate concentration on the expression of IIS and TOR transcriptionally regulated genes in females and males. Surfaces show the relationship between gene expression, carbohydrate level and protein level in female and male flies. Expression of (A, A′) InR, and (B, B′) 4E-BP, both negatively regulated by the activity of the IIS via the Forkhead transcription factor FOXO. (C, C′) Expression of Ash2L, ostensibly negatively regulated by the activity of TOR signaling. (D, D′) Expression of CG3071, ostensibly positively regulated by the activity of TOR signaling. Points indicate diets tested and dotted lines connect diets with equal protein-to-carbohydrate ratios (1:16, 1:8, 1:4, 1:2, 1:1, 2:1). [file 13227_2021_175_MOESM2_ESM.pdf]

♀

A

*dILP2*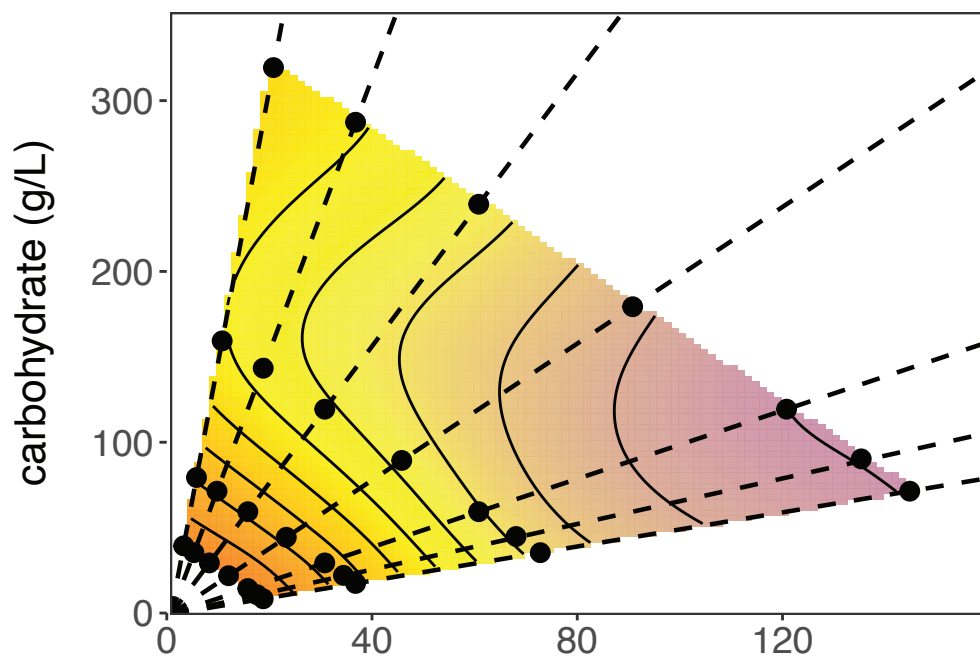

♂

A' *dILP2*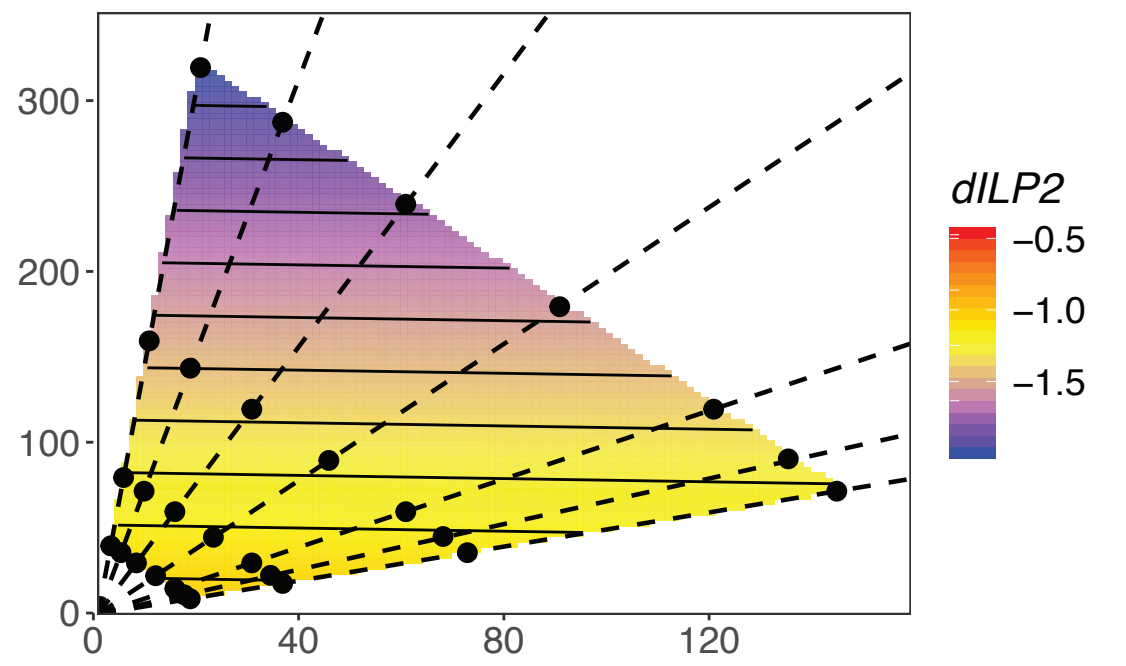

B

*dILP3*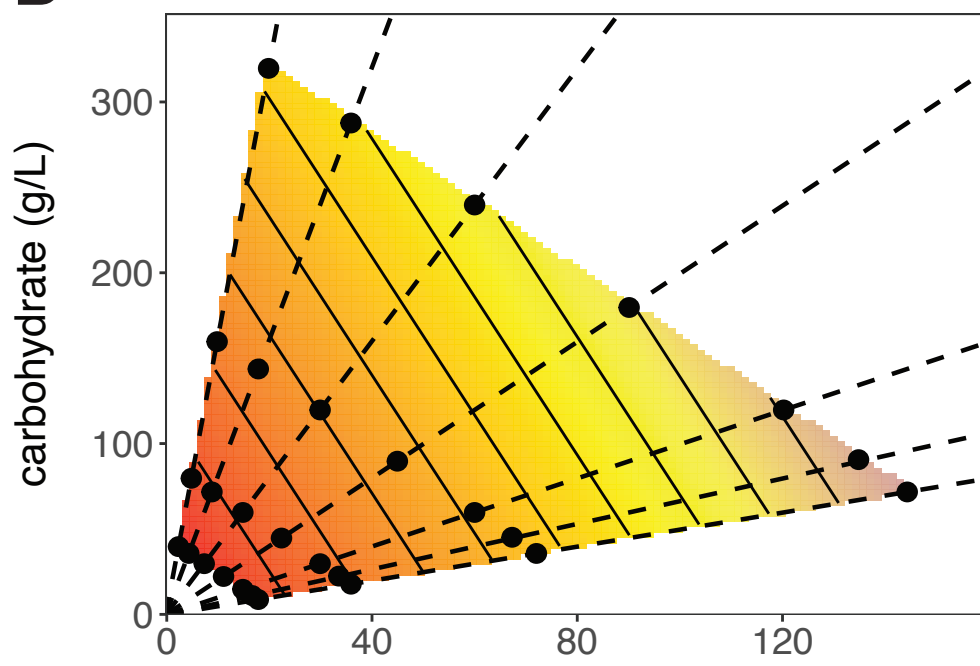B' *dILP3*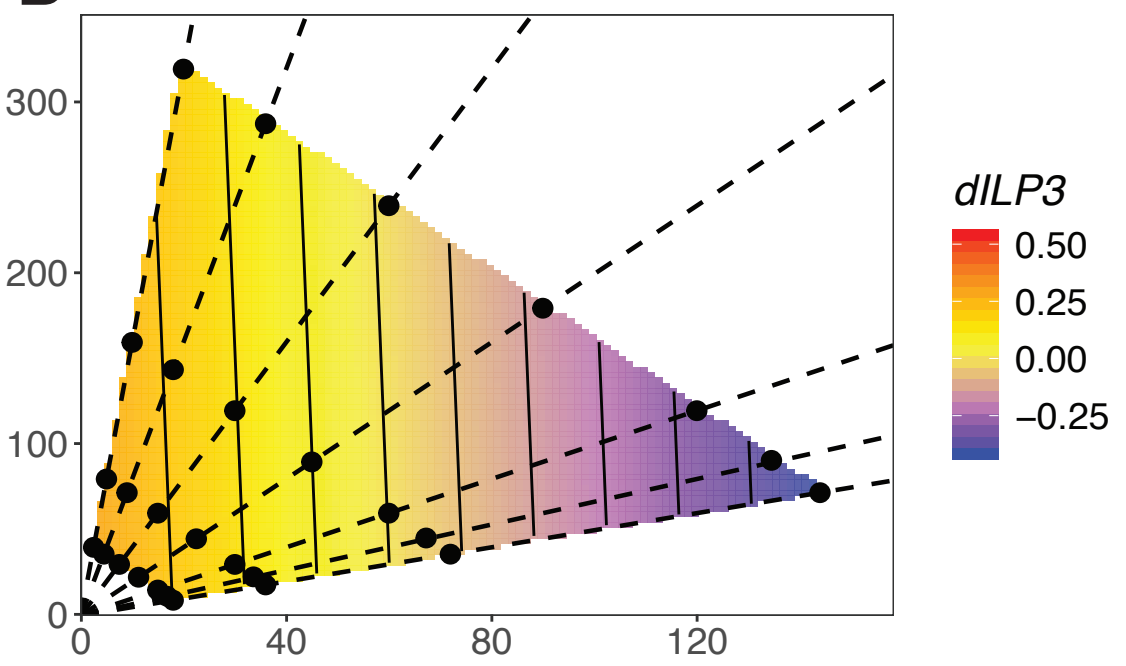

C

*dILP5*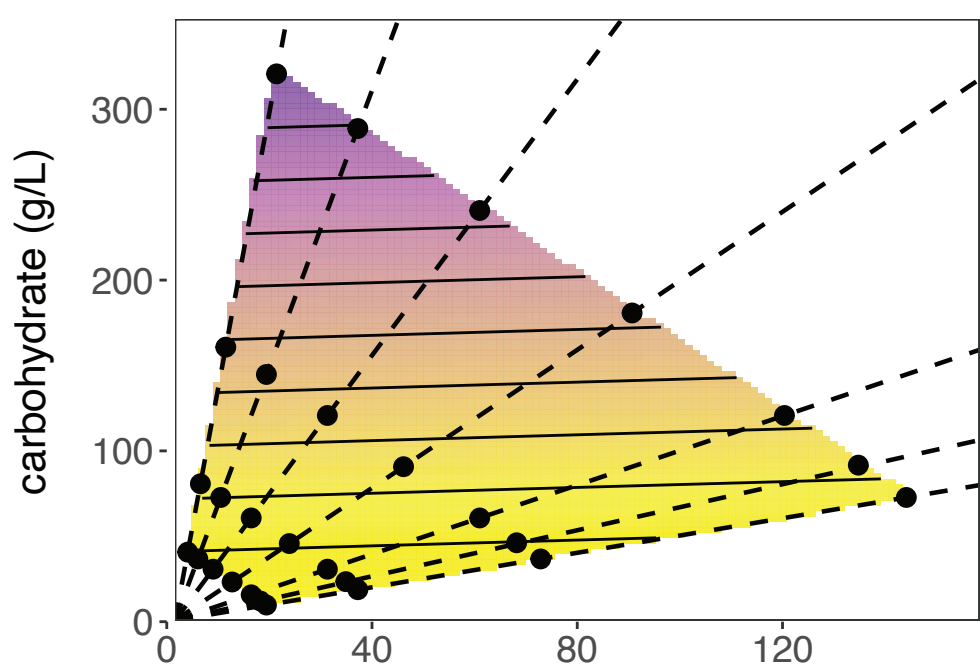C' *dILP5*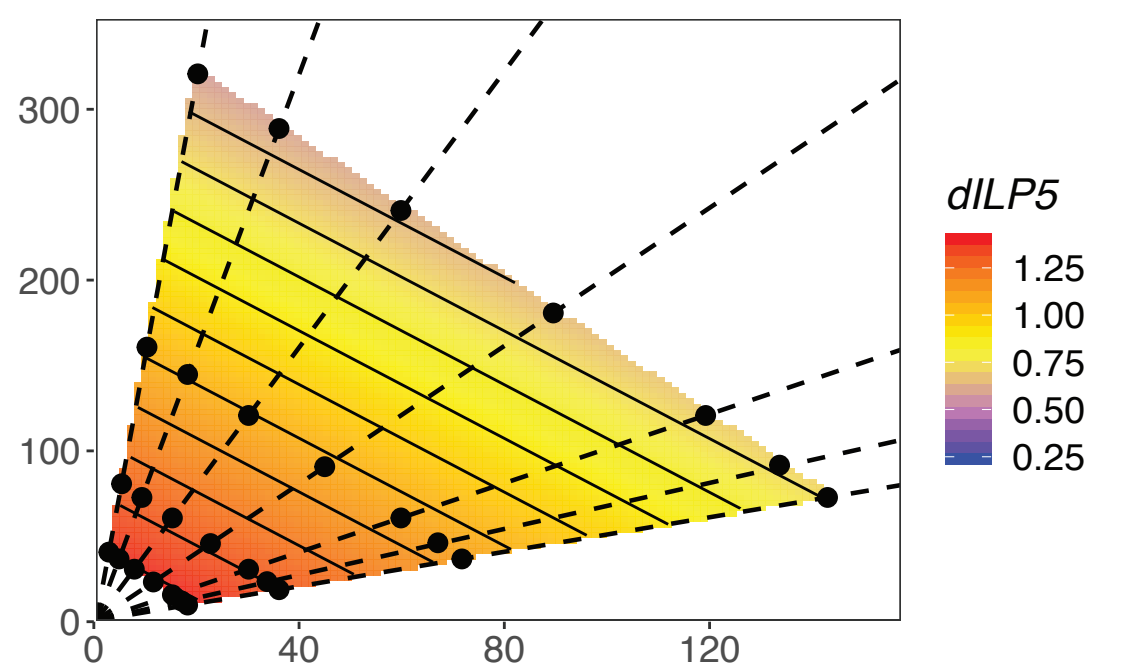

D

*dILP8*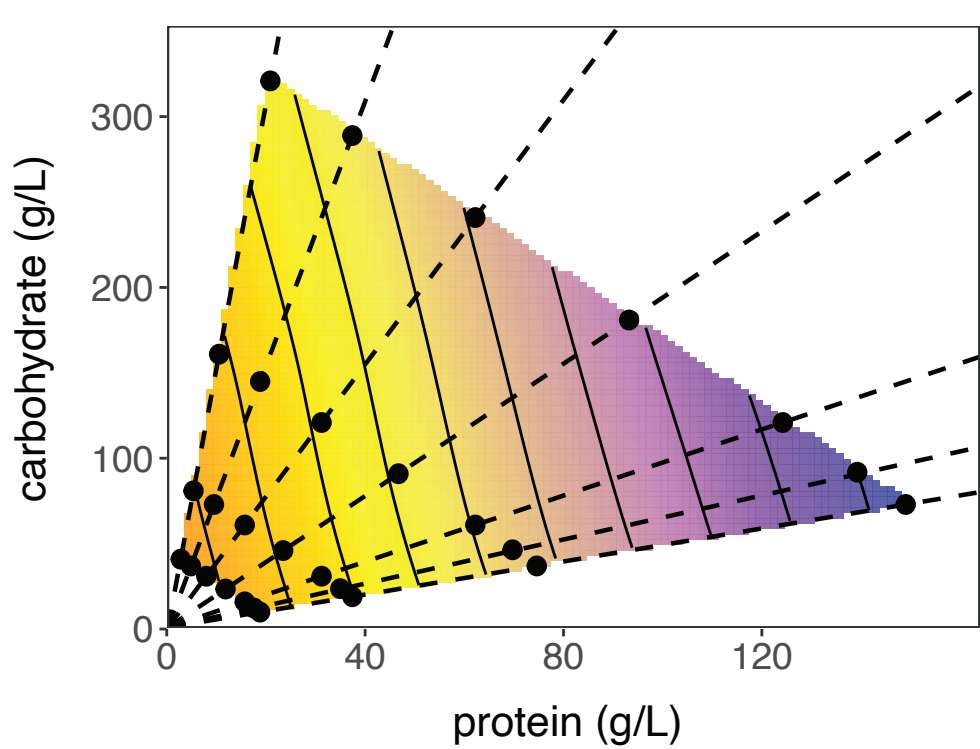D' *dILP8*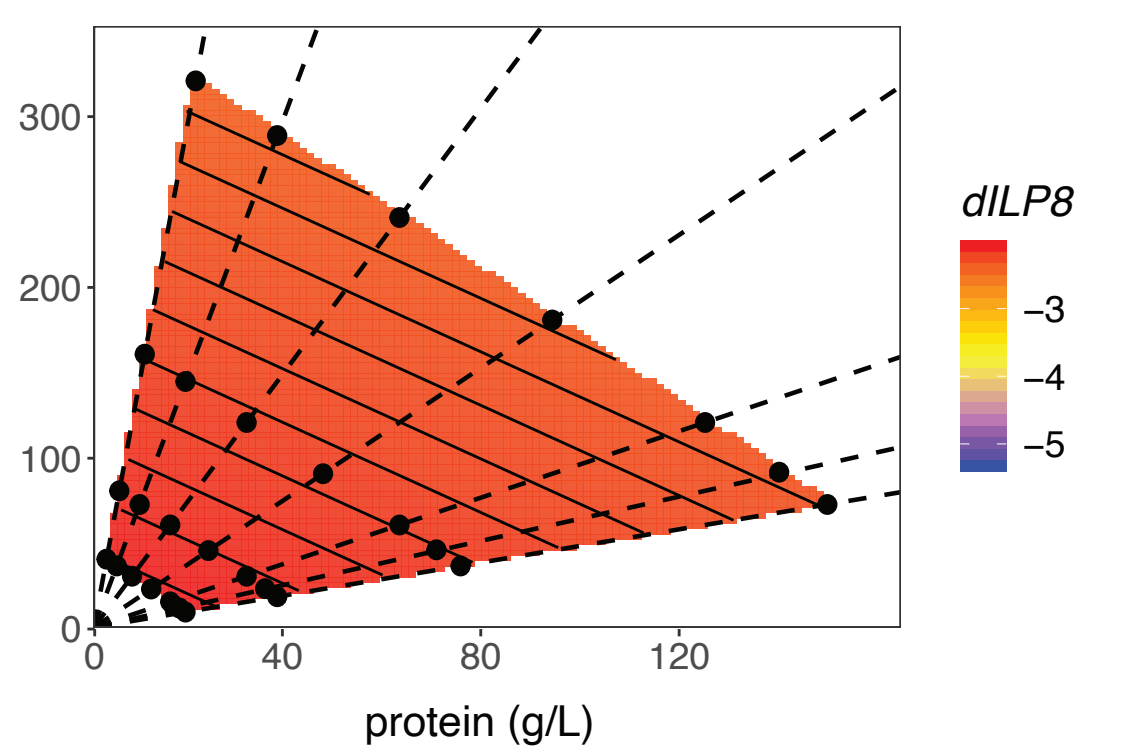

Supplement: Supplementary file 3 — Additional file 3: Fig. S3. Thin plate spline of the effect of protein and carbohydrate concentration on the expression of dILPs in females and males. Surfaces show the relationship between gene expression, carbohydrate level and protein level in female and male flies. (A) dILP2. (B) dILP3. (C) dILP 5. (D) dILP8. Points indicate diets tested and dotted lines connect diets with equal protein-to-carbohydrate ratios (1:16, 1:8, 1:4, 1:2, 1:1, 2:1). Corresponding thin-plate spline plots are shown in Additional file 1: Fig. S1. [file 13227_2021_175_MOESM3_ESM.pdf]
