# Supplementary material for: Sex-specific plasticity and the nutritional geometry of insulin-signaling gene expression in Drosophila melanogaster
Source: EvoDevo. 2021 May 14;12:6. doi: 10.1186/s13227-021-00175-0 (PMC8120840; doi:10.1186/s13227-021-00175-0)
Supplement: Supplementary file 4 — Additional file 4: Table S1. Variation in gene expression among samples within diets for male and female samples. [file 13227_2021_175_MOESM4_ESM.docx]

**Supplementary Table 1: Variation in gene expression among samples within diets for male and female samples.**

|  | *4E-BP* | *InR* | *Ash2L* | *CG3071* | *dILP2* | *dILP3* | *dILP5* | *dILP8* |
| --- | --- | --- | --- | --- | --- | --- | --- | --- |
| Female  RMSE^A^ | 0.4184 | 0.5834 | 0.5233 | 0.5070 | 0.0505 | 0.4012 | 0.7208 | 1.3167 |
| Male  RMSE^A^ | 0.5041 | 0.6971 | 0.6720 | 0.4876 | 1.4900 | 1.2494 | 0.9775 | 1.0351 |
| *P*-Value^B^ | 0.2752 | 0.3739 | 0.1016 | 0.1628 | **<0.0001** | **<0.0001** | **0.0389** | **0.0018** |

^A^ Root mean square error from the model *T = D* where *T* is expression level of gene and *D* is diet (protein and carbohydrate combination).

^B^ *P-*value for *F* test comparing residual variance for males and female level of gene expression, after fitting the model *T = D*. Significant P-values are shown in bold
